# Associations of free, bioavailable and total 25-hydroxyvitamin D with neonatal birth anthropometry and calcium homoeostasis in mother–child pairs in a sunny Mediterranean region

**DOI:** 10.1017/S000711452300243X

**Published:** 2024-03-14

**Authors:** Hana M. A. Fakhoury, Tarek Ziad Arabi, Hani Tamim, Rene F. Chun, William B. Grant, Martin Hewison, Fatme AlAnouti, Stefan Pilz, Cedric Annweiler, Georgios Tzimagiorgis, Costas Haitoglou, Spyridon N. Karras

**Affiliations:** 1 Department of Biochemistry and Molecular Medicine, College of Medicine, Alfaisal University, Riyadh, Saudi Arabia; 2 Department of Orthopaedic Surgery, David Geffen School of Medicine at UCLA, Los Angeles, CA, USA; 3 Sunlight, Nutrition, and Health Research Center, P.O. Box 641603, San Francisco, CA 94164-1603, USA; 4 Institute of Metabolism and Systems Research, University of Birmingham, Birmingham, UK; 5 Department of Public Health and Nutrition, College of Natural and Health Sciences, Zayed University, Abu Dhabi, United Arab Emirates; 6 ASPIRE Precision Medicine Research Institute Abu Dhabi, United Arab Emirates; 7 Division of Endocrinology and Diabetology, Department of Internal Medicine, Medical University of Graz, Graz, Austria; 8 UNIV ANGERS, UPRES EA 4638, University of Angers, Department of Geriatric Medicine and Memory Clinic, Research Center on Autonomy and Longevity, Angers University Hospital, Angers, France; 9 Laboratory of Biological Chemistry, Medical School, Aristotle University, 54636 Thessaloniki, Greece

**Keywords:** Vitamin D, Neonatal, Bioavailable, Anthropometry

## Abstract

Sufficient vitamin D status is crucial for successful pregnancy and fetal development. The assessment of 25-hydroxyvitamin D (25(OH)D) concentrations is commonly used to evaluate vitamin D status. Our objective was to examine the interrelated biodynamics of maternal and neonatal total, free and bioavailable 25(OH)D in maternal–neonatal dyads at birth and their associations with homeostasis and neonatal birth anthropometry. We analysed a cohort of seventy full-term mother–child pairs. We found positive associations between all neonatal measures of vitamin D status. Maternal forms exhibited a similar pattern of association, except for the bioavailable maternal form. In multivariate analysis, both total and free maternal 25(OH)D concentrations were correlated with all neonatal forms (neonatal total 25(OH)D: 1·29 (95 % CI, 1·12, 1·46) for maternal total 25(OH)D, 10·89 (8·16, 13·63) for maternal free 25(OH)D), (neonatal free 25(OH)D: 0·15 for maternal total 25(OH)D, 1·28 (95 % CI, 0·89, 1·68) for maternal free 25(OH)D) and (0·13 (95 % CI, 0·10, 0·16), 1·06 (95 % CI, 0·68, 1·43) for maternal free 25(OH)D), respectively, with the exclusion of the bioavailable maternal form. We observed no significant interactions within or between groups regarding maternal and neonatal vitamin D parameters and maternal calcium and parathyroid hormone concentrations, and neonatal birth anthropometry. Our study indicates that bioavailable maternal and neonatal 25(OH)D have no significant effects on vitamin D equilibrium, Ca homeostasis and neonatal anthropometry at birth. However, we observed an interaction between maternal and neonatal total and free 25(OH)D concentrations at the maternal–neonatal interface, with no associations observed with other calciotropic or anthropometric outcomes.

During pregnancy, maternal metabolism undergoes significant changes to accommodate the increased fetal demands^(1)^. Among such changes is the increase in 1,25-dihydroxyvitamin D, in early pregnancy until delivery^(2)^. Vitamin D metabolites are key players in fetal cellular regulation and antimicrobial protection at the uterine decidua^(3)^. Therefore, maternal vitamin D deficiency may have several adverse impacts on pregnancy outcomes and has been correlated with pre-eclampsia, gestational diabetes, preterm birth and low birthweight^(4)^. Furthermore, it has been suggested that a poor prenatal vitamin D status can increase the risk of various adulthood diseases, including insulin-dependent diabetes mellitus, multiple sclerosis, schizophrenia and cancers^(5)^. However, available results regarding these implications vary largely due to the diversity of study designs, patient populations and geographical region^(1)^.

Although total 25-hydroxyvitamin D (25(OH)D) is the most common measurement of vitamin D status, there is a large debate regarding the most accurate parameter of vitamin D status in certain conditions such as pregnancy^(1)^. Circulating 25(OH)D can be found in three forms: free, weakly bound to albumin or bound to vitamin D-binding protein (VDBP)^(6)^. VDBP concentrations increase significantly in pregnancy, impacting all vitamin D metabolites and their physiologic activity^(7)^. Low concentrations of maternal VDBP have been linked with endometriosis, infertility, gestational diabetes, pre-eclampsia and fetal growth restriction^(7)^.

According to the ‘free hormone hypothesis’, free vitamin D metabolites are the only physiologic active types of vitamin D metabolites due to their lipophilic activity^(8)^. Hence, studies have aimed to determine whether free vitamin D metabolite concentrations may provide a more reliable marker of vitamin D status. However, a study assessing 241 Spanish children found no benefit for free 25(OH)D measurement over total 25(OH)D^(9)^. In pregnant adolescents, Best *et al*. found that total 25 (OH)D concentrations correlated more strongly than free 25(OH)D with parathyroid hormone (PTH) and 24,25(OH)_2_D concentrations^(10)^. Contrarily, Tsuprykov*et al*. demonstrated that free 25(OH)D showed stronger associations with bone and lipid biomarkers than total 25(OH)D^(11)^. Hence, further studies are needed to determine the clinical utility of total or free 25(OH)D concentrations.

Studies assessing the correlation between free and bioavailable (free plus albumin-bound) 25(OH)D concentrations with pregnancy outcomes and neonatal parameters remain limited.

A study by Fernando *et al.* retrospectively assessed the association of free and total 25(OH)D concentrations with neonatal outcomes in 304 women^(1)^. The authors found that total, free, but not bioavailable, maternal 25(OH)D concentrations were significantly correlated with neonatal birthweight. Our study aimed to evaluate the interrelated biodynamics of maternal and neonatal total, free and bioavailable 25(OH)D of maternal–neonatal dyads at birth and their potential associations with Ca homoeostasis and neonatal birth anthropometry, in a sunny Mediterranean region.

## Methods

### Participants

This an extension of a previous study of maternal–neonatal pairs, including new participants with the same study protocol. In this study, data were collected from seventy mother–child pairs at birth, as described previously^(12)^. All the women in the study had fair skin and met the inclusion criterion of having a full-term pregnancy between 37 and 42 gestational weeks. Maternal exclusion criteria included primary hyperparathyroidism, secondary osteoporosis, heavy alcohol use, hyperthyroidism, nephritic syndrome, inflammatory bowel disease, rheumatoid arthritis, osteomalacia, obesity, gestational diabetes and the use of medications affecting Ca or vitamin D status. Neonatal exclusion criteria included being small-for-gestational age and having serious congenital anomalies. This study was conducted according to the guidelines laid down in the Declaration of Helsinki, and all procedures were approved by the Bioethics Committee of the Aristotle University of Thessaloniki, Greece (initial approval number 1/19-12-2011, extension 2/6-6-2022). Written informed consent was obtained from all mothers included in the study.

### Demographic data – biochemical and hormonal assays

At enrollment, maternal demographic and social characteristics were recorded (age, weight pre-pregnancy, weeks of gestation and previous live births). Maternal alcohol use during pregnancy was defined either as none (subdivided into never drinking alcohol or drinking alcohol but not during pregnancy), light (1–2 units per week or at any one-time during pregnancy) or moderate (3–6 units per week or at any one-time during pregnancy). Maternal education was classified as standard (secondary) and higher (tertiary and holding of academic degrees). Maternal blood samples were collected 30–60 min before delivery, and umbilical cord blood was collected after clamping. Biochemical analysis for Ca, phosphorus, albumin, PTH, 25(OH)D_2_ and 25(OH)D_3_ was performed according to methods described previously^(12,13)^. Concentrations of,25(OH)D_2_ and 25(OH)D_3_ were measured using liquid chromatography–tandem mass spectrometry, with lower limits of quantification of 0·5 ng/ml for each form. Total 25(OH)D was determined by summing the concentrations of both forms.^(12,13)^. Briefly, the assay involves a chiral column in tandem with a rapid resolution microbore column along with liquid–liquid extraction. The method is fully validated using quality controls at four different concentration levels. Quality controls were calculated after chromatographically separating the epimers, isobars and other analogues. The same concentrations were recovered from spiked quality controls prepared in house. The accuracy of the assay was also double checked using DEQAS and Chromsystem quality controls. Full method validation parameters have been reported previously.^(14–16)^

PTH determinations were performed using the electrochemiluminescence immunoassay ECLIA (Roche Diagnostics GmbA). Reference range for PTH was 15–65 pg/ml, functional sensitivity 6·0 pg/ml, within-run precision 0·6–2·8 % and total precision 1·6–3·4 %.

Corrected Ca was calculated using the following formula: corrected Ca (mg/dl) = (0·8 × (normal albumin - patient’s albumin)) + serum Ca. VDBP was measured with the quantitative sandwich enzyme immunoassay technique (R&D System). Intra- and inter-assay coefficient of variation was 1·3 and 2·2 %, respectively.

### Estimation of free and bioavailable vitamin D

Values for free and bioavailable 25(OH)D in mothers and neonates were estimated using a mathematical calculation based on the previously published work of Chun et al.^(17)^ To provide a brief overview, the measured values of VDBP (μM), ALB (μM), 25(OH)D (nM) and 1,25(OH)2D (nM) in the subject samples were inputted into a MATLAB script that implements the mathematical model described by Chun *et al.* Serum-free 25(OH)D levels were converted from nM to pg/ml by multiplying 0·4166 × 10^3.

This script generates output estimations of free and bioavailable 25(OH)D. If interested, the script is available upon request from R. Chun or B. E. Peercy.

### Maternal and neonatal anthropometric data

Maternal (body weight) and neonatal anthropometric characteristics were recorded at enrollment, and umbilical cord blood samples were stored at −70°C until assays were performed. Neonatal anthropometry was evaluated within 72 h of birth, and measurements were taken by the same trained nurse using standard techniques^(13)^. Birth weight, head circumference, chest circumference and abdominal circumference were all measured. Birth weight was calculated as the mean of ten sequential readings obtained from calibrated scales. The maximum head, chest and abdominal circumferences were measured using a plastic encircling tape.

### Statistical analysis

Data were entered into an Excel sheet, which was then transferred into the Statistical Package for Social Sciences (SPSS, version 28, IBM Corp.), which was used for data cleaning, management and analysis. Descriptive statistics were carried out, and results were reported as number and percent for categorical variables, whereas mean and standard deviation were reported for continuous ones. To assess the associations between different variables, univariate and multivariate linear regression analysis was performed, where baseline factors were considered as potential confounders (mainly, age, BMI and previous live birth)^(1,18)^. Results were reported as beta coefficient (*β*) and 95 % CI. Moreover, scatter plots were constructed to assess the associations between different variables. To adjust for multiple comparisons, Bonferroni’s correction was used, where the *P* value was adjusted by dividing the error by 2 (since two major sets of analyses were done, for maternal and for neonatal), and thus a *P* value of < 0·025 was used to indicate statistical significance.

## Results

### Patient characteristics

The mean maternal age was 31·9 ± 6·1 years, and the mean gestational age at birth was 38·8 ± 1·6 weeks (Table 1).The average maternal pre-pregnancy and term weights were 67·6 ± 14·5 and 81·4 ± 14·3 kg, respectively. Approximately two-thirds of the patients (67·7 %) were null gravid, and the vast majority of patients did not smoke or drink alcohol during pregnancy. The average levels of maternal and neonatal serum 25(OH)D levels are presented in Table 2.

**Table 1.** Characteristics of the study population

	Mean	sd
Age (years)	32	6
Weight pre-pregnancy (kg)	68	15
Weight term (kg)	81	14
Weeks of gestation	38·8	1·6
	*n*	%
Previous live births		
0	44	63
1	11	16
2	8	11
3	1	1
4	1	1
Missing	5	7
Educational level		
1	52	74
2	14	20
Missing data	4	6
Alcohol consumption during pregnancy		
None	58	83
Yes	8	11
Missing data	4	6
Smoking during pregnancy		
None	56	80
Yes	10	14
Missing data	4	6

**Table 2.** Mean serum levels of total, free and bioavailable 25(OH)D in mothers and neonates

Serum parameter	Maternal levels		Neonatal levels		*P* value
	Mean	sd	Mean	sd	
Total (nM)	30·48	17·46	41·76	23·88	0·003
Bioavailable (nM)	1·61	1·06	3·53	2·62	< 0·0001
Free (pg/ml)	2·63	1·72	4·22	3·01	< 0·0001
VDBP (µg/ml)	364·09	98·45	355·25	233·4	0·78
Albumin (g/dl)	2·93	0·47	3·78	0·6	< 0·0001

25(OH)D, 25-hydroxyvitamin; VDBP, vitamin D-binding protein.

### Interrelations between vitamin D forms in maternal–neonatal dyads

When comparing all three forms of maternal 25(OH)D, a significant positive association was found between total and free 25(OH)D (*β* = 8·36 (95 % CI, 6·98, 9·75)). However, no significant associations were observed between bioavailable 25(OH)D with free and total forms of 25(OH)D (Table 3).On the other hand, all forms of neonatal 25(OH)D (total, free and bioavailable) were positively interrelated (Table 3). Specifically, there was a significant positive association between total and free 25(OH)D (*β* = 6·10 (95 % CI, 4·92, 7·28)), as well as between total 25(OH)D and bioavailable 25(OH)D (*β* = 6·48 (95 % CI, 4·92, 8·04)).

**Table 3.** Maternal–maternal and neonatal–neonatal interrelations between vitamin D forms

Maternal							Neonatal						
	Total 25(OH)D (nM)	95 % CI	Free 25(OH)D (pg/ml)	95 % CI	Bioavailable 25(OH)D (nM)	95 % CI		Total 25(OH)D (nM)	95 % CI	Free 25(OH)D (pg/ml)	95 % CI	Bioavailable 25(OH)D (nM)	95 % CI
Maternal total 25(OH)D (nM)	NA		8·36	6·98, 9·75	1·05	–3·08, 5·18	Neonatal total 25(OH)D (nM)	NA		6·10	4·92, 7·28	6·48	4·92, 8·04
			< 0·001		0·61					< 0·001		< 0·001	
Maternal free 25(OH)D (pg/ml)	0·09	0·07, 0·10	NA		–0·09	–0·52, 0·34	Neonatal-free 25(OH)D (pg/ml)	0·11	0·09, 0·13	NA		1·08	0·97, 1·19
	< 0·001				0·67			< 0·001				< 0·001	
Maternal bioavailable 25D (nM)	0·00	–0·01, 0·02	–0·04	–0·21, 0·14	NA		Neonatal bioavailable 25(OH)D (nM)	0·09	0·07, 0·11	0·82	0·73, 0·90	NA	
	0·61		0·67					< 0·001		< 0·001			

25(OH)D, 25-hydroxyvitamin

### Maternal–neonatal vitamin D relations

**Fig. 1.**
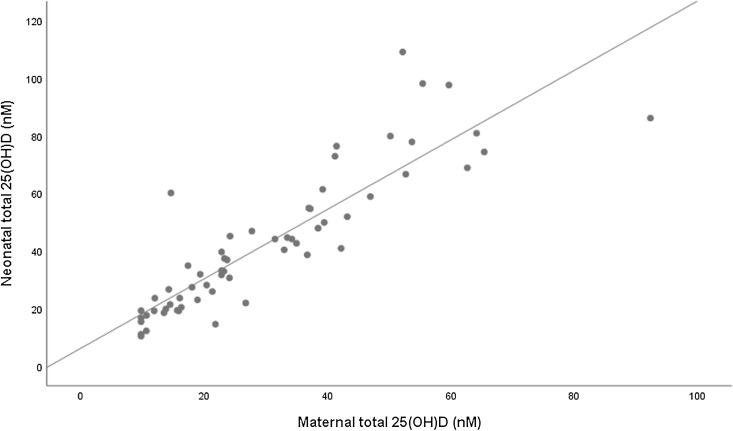
Correlation between maternal total 25(OH)D (nM) and neonatal total 25 (OH)D (nM). R = 0·879, *P* < 0·001, total 25(OH)D neonate = 1·204 * total 25(OH)D mother + 6·337. 25(OH)D, 25-hydroxyvitamin

**Fig. 2.**
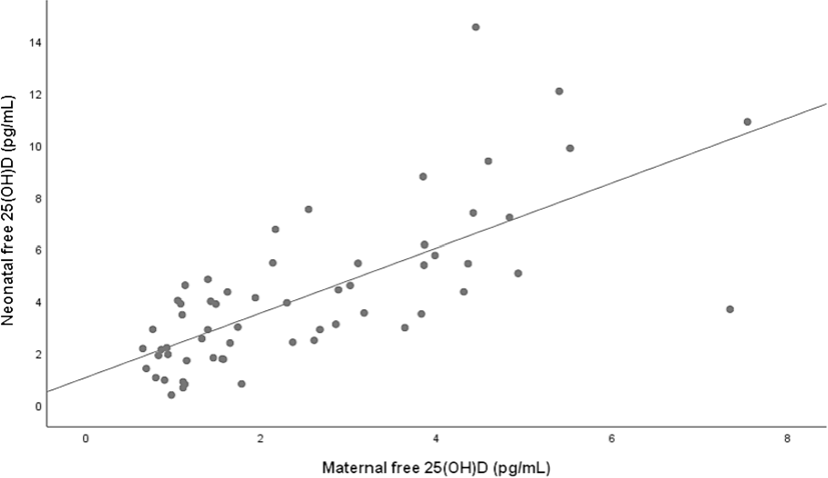
Correlation between maternal free 25(OH)D (pg/ml) and neonatal free 25OHD (pg/ml). R = 0·711, *P* < 0·001, free 25(OH)D neonate = 1·245 * free 25 (OH)D mother + 1·063. 25(OH)D, 25-hydroxyvitamin

**Fig. 3.**
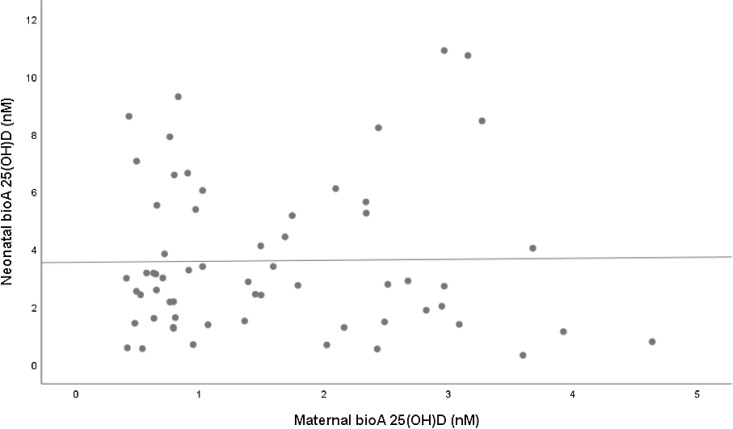
Correlation between maternal bioavailable 25D(OH)D (nM) and neonatal bioavailable 25(OH)D (nM). R = 0·14, *P* = 0·914, BioA 25(OH)D neonate = 0·035 * BioA 25(OH)D mother + 3·547. 25(OH)D, 25-hydroxyvitamin

Table 4 shows the results of univariate and multivariate analyses of the association between maternal 25(OH)D concentrations (total, free and bioavailable) and neonatal 25(OH)D concentrations (total, free and bioavailable). Both maternal free and total 25(OH)D forms were independently associated with all neonatal vitamin D forms (Fig. 1 and 2). Specifically, maternal 25(OH)D showed a strong positive correlation with neonatal total vitamin D (*β* = 10·89 (8·16, 13·63)). However, there was no association between maternal bioavailable vitamin D and the neonatal forms (Fig. 3).

**Table 4.** Regression analysis between maternal-neonatal vitamin D forms

	Maternal total 25(OH)D (nM)				Maternal free 25(OH)D (pg/ml)				Maternal bioavailable25(OH)D (nM)				Maternal VDBP µg/ml			
	Univariate		Multivariate		Univariate		Multivariate		Univariate		Multivariate		Univariate		Multivariate	
	*β*	95 % CI	*β*	95 % CI	*β*	95 % CI	*β*	95 % CI	*β*	95 % CI	*β*	95 % CI	*β*	95 % CI	*β*	95 % CI
Neonatal total 25(OH)D (nM)	1·20	1·03, 1·37	1·29	1·12, 1·46	10·87	8·39, 13·35	10·89	8·16, 13·63	2·23	–3·64, 8·10	1·97	–4·20, 8·13	–0·01	–0·08, 0·05	–0·01	–0·08, 0·05
	< 0·001		< 0·001		< 0·001		< 0·001		0·45		0·52		0·64		0·68	
Neonatal free 25(OH)D (pg/ml)	0·14	0·11, 0·17	0·15	0·12, 0·19	1·25	0·92, 1·57	1·28	0·89, 1·68	0·17	–0·56, 0·91	0·25	–0·57, 1·08	–0·003	–0·011, 0·005	–0·004	–0·012, 0·005
	< 0·001		< 0·001		< 0·001		< 0·001		0·64		0·54		0·52		0·43	
Neonatal bioavailable 25(OH)D (nM)	0·12	0·09, 0·14	0·13	0·10, 0·16	1·01	0·69, 1·32	1·06	0·68, 1·43	0·03	–0·61, 0·68	0·06	–0·66, 0·79	–0·001	–0·008, 0·006	–0·003	–0·010, 0·005
	< 0·001		< 0·001		< 0·001		< 0·001		0·91		0·86		0·71		0·51	
Neonatal VDBP µg/ml	–1·95	–5·20, 1·31	–2·43	–6·41, 1·56	–21·74	–56·74, 13·27	–29·44	–70·82, 11·95	9·16	–45·15, 63·48	10·30	–50·64, 71·24	0·19	–0·43, 0·82	0·34	–0·35, 1·02
	0·24		0·23		0·22		0·16		0·74		0·74		0·53		0·33	

25(OH)D, 25-hydroxyvitamin D; VDBP, vitamin D-binding protein

### Maternal vitamin D forms, serum parathyroid hormone and calcium

Among maternal 25 (OH)D parameters, only total 25(OH)D had a borderline effect on maternal corrected Ca concentrations (*β* = 0·01 (0, 0·01)). Maternal PTH was negatively correlated with free (*β* = –2·43 (–4·63, −0·23)) and total maternal 25(OH)D (0·34 (–0·55, −0·13)). Furthermore, maternal 25(OH)D parameters were not independently associated with neonatal corrected Ca and PTH concentrations. Table 5 illustrates the results of univariate and multivariate analyses examining the association between neonatal 25(OH)D concentrations (total, free and bioavailable) and neonatal corrected Ca and serum PTH concentrations. Neonatal bioavailable 25(OH)D showed a borderline effect on neonatal corrected Ca concentrations (*β* = 0·069 (0·001, 0·136)). Other 25(OH)D forms were not associated with neonatal corrected Ca. Furthermore, all neonatal forms demonstrated no effect on neonatal PTH.

**Table 5.** Regression analysis between neonatal vitamin D forms and neonatal serum PTH and calcium

	Neonatal total 25(OH)D (nM)				Neonatal free 25(OH)D (pg/ml)				Neonatal bioavailable 25(OH)D (nM)			
	Univariate		Multivariate		Univariate		Multivariate		Univariate		Multivariate	
	*β*	95 % CI	*β*	95 % CI	*β*	95 % CI	*β*	95 % CI	*β*	95 % CI	*β*	95 % CI
Neonatal corrected Ca (mg/dl)	–0·002	–0·010, 0·006	–0·001	–0·010, 0·007	0·004	–0·061, 0·070	0·01	–0·05, 0·07	0·07	0·00, 0·15	0·069	0·001, 0·136
	0·66		0·74		0·90		0·68		0·049		0·046	
Neonatal serum parathyroid hormone (pg/ml)	–0·01	–0·03, 0·01	–0·02	–0·04, 0·01	–0·09	–0·25, 0·07	–0·11	–0·29, 0·07	–0·12	–0·30, 0·06	–0·14	–0·35, 0·06
	0·33		0·21		0·25		0·25		0·19		0·17	

PTH, parathyroid hormone; 25(OH)D, 25-hydroxyvitamin D.

### Maternal and neonatal vitamin D forms and neonatal outcomes

No significant associations were observed between all maternal 25(OH)D forms and neonatal outcome measures, including birth weight and head, chest and abdomen circumference. Similarly, there was no relation between neonatal 25(OH)D and neonatal outcomes.

## Discussion

The aim of this study was to investigate the relationship between various measures of 25 (OH)D status in maternal and neonatal populations, as well as their associations with neonatal outcomes. The results showed a positive correlation between maternal total and free 25(OH)D concentrations, while all forms of neonatal 25(OH)D were positively interrelated. Neonatal outcomes were not significantly influenced by maternal and neonatal 25(OH)D forms, with the exception of a mild effect of neonatal bioavailable 25(OH)D on neonatal Ca. Multivariate analysis revealed that maternal total and free 25(OH)D concentrations were correlated with all neonatal forms, but not with maternal bioavailable 25(OH)D. This suggests that maternal total and free 25(OH)D may play a more important role in influencing neonatal vitamin D status than maternal bioavailable 25(OH)D. However, the lack of association between maternal total and bioavailable 25(OH)D requires further interpretation. Regarding serum PTH concentrations, there was no significant association between the three forms of 25(OH)D and serum PTH concentrations in neonates. However, neonatal bioavailable 25(OH)D showed a borderline association with corrected neonatal Ca concentrations, indicating a possible minor effect on neonatal Ca concentrations.

Several previous studies have suggested that relying solely on total 25(OH)D concentration may not be sufficient in certain cases like pregnancy, liver cirrhosis, use of hormonal contraceptives and kidney disease, where VDBP concentrations may be altered^(19)^. This can lead to decreased bioavailable or free 25(OH)D concentrations, which may be a more appropriate measure of vitamin D status in these populations. A Korean study evaluated the usefulness of bioavailable 25(OH)D when VDPB concentrations were altered by pregnancy or liver cirrhosis^(20)^. Patients with liver cirrhosis had significantly lower VDBP and total 25(OH)D concentrations compared with healthy controls, while pregnant women had significantly higher VDBP concentrations. Although total 25(OH)D concentrations in pregnant women were similar to those in controls, their bioavailable 25(OH)D concentrations were significantly lower. Another German study found that free 25(OH)D was lower in the third trimester of pregnancy compared with the first trimester, while total 25(OH)D was not decreased in late pregnancy^(21)^. An observational study conducted in Brazil found that serum 25(OH)D was inversely associated with the gestational week and season during pregnancy, with lower levels in autumn and winter^(22)^.

Bikle and Schwartz^(23)^ reviewed the free hormone hypothesis for vitamin D and suggested that only free 25(OH)D can enter cells. However, calculating free 25(OH)D from measurements of 25(OH)D and VDBP has proven difficult, as many clinical conditions can alter the relationship. Therefore, measuring bioavailable or free 25(OH)D might be more advantageous in certain populations. A study of vitamin D status among women was conducted in Slovenia^(24)^. The vitamin D status was determined by measuring the total 25(OH)D concentration, DBP and albumin and calculating the bioavailable 25(OH)D and free 25(OH)D. For the calculation of bioavailable and free 25(OH)D, a new online calculator was developed. Premenopausal women had 12 % lower total 25(OH)D, 32 % lower bioavailable 25(OH)D and 25 % higher DBP than postmenopausal women. In postmenopausal women, the measurement of free or bioavailable 25(OH)D instead of the total 25(OH)D could be advantageous.

Several studies have found a positive association between maternal and neonatal total 25(OH)D levels^(25–27)^. Hence, maternal vitamin D status could significantly impact neonatal vitamin D status at birth. Maternal vitamin D deficiency places neonates at an increased risk of vitamin D deficiency by approximately 17-fold^(28)^. In line with these findings, our study revealed that maternal total 25(OH)D was independently associated with all forms of neonatal 25(OH)D. Similarly, maternal free 25(OH)D was independently associated with neonatal forms of vitamin D; however, no significant association was seen with maternal bioavailable 25(OH)D. To our knowledge, no other reports of maternal–neonatal vitamin D relationships are available in the literature from the Mediterranean region.

Studies assessing the effects of maternal bioavailable and free vitamin D on neonatal vitamin D parameters and outcomes are extremely limited. The relationship between maternal total 25(OH)D and neonatal birth weight remains controversial. Although several studies have revealed the existence of a positive association between total 25(OH)D concentrations and birthweight^(28–31)^, numerous other investigators have reported no such correlation^(32,33)^. Fernando *et al.* previously demonstrated that total and free 25(OH)D were independently associated with neonatal birthweight^(1)^. By contrast, we found no association between maternal 25(OH)D and birthweight. Further studies are needed to confirm the role of maternal vitamin D status for neonatal outcomes.

The main limitation of our study stems from the fact that this is a single-centre study that limits the generalisability of our data. It is well known that vitamin D levels fluctuate greatly across geographical locations and seasons, and such variables were not taken into consideration in the present study^(1)^. Additionally, due to similar results relating to total and free 25(OH)D, we were unable to determine which parameter is more clinically relevant, and it remains unclear whether free 25(OH)D provides superior insight into maternal and neonatal vitamin D status.

In conclusion, the findings of this current study suggest that maternal total and free 25(OH)D concentrations may play a more important role than maternal bioavailable 25(OH)D in influencing neonatal vitamin D status. More data are needed to support the importance of measuring the various forms of 25(OH)D to assess vitamin D status in mothers and their neonates.
